# Analgesic and Anti-Inflammatory Properties of Gelsolin in Acetic Acid Induced Writhing, Tail Immersion and Carrageenan Induced Paw Edema in Mice

**DOI:** 10.1371/journal.pone.0135558

**Published:** 2015-08-14

**Authors:** Ashok Kumar Gupta, Devraj Parasar, Amin Sagar, Vikas Choudhary, Bhupinder Singh Chopra, Renu Garg, Ashish, Neeraj Khatri

**Affiliations:** 1 Division of Animal Facility, Council of Scientific and Industrial Research—Institute of Microbial Technology, Chandigarh, India; 2 Division of Protein Science & Engineering, Council of Scientific and Industrial Research-Institute of Microbial Technology, Chandigarh, India; Temple University, UNITED STATES

## Abstract

Plasma gelsolin levels significantly decline in several disease conditions, since gelsolin gets scavenged when it depolymerizes and caps filamentous actin released in the circulation following tissue injury. It is well established that our body require/implement inflammatory and analgesic responses to protect against cell damage and injury to the tissue. This study was envisaged to examine analgesic and anti-inflammatory activity of exogenous gelsolin (8 mg/mouse) in mice models of pain and acute inflammation. Administration of gelsolin in acetic acid-induced writhing and tail immersion tests not only demonstrated a significant reduction in the number of acetic acid-induced writhing effects, but also exhibited an analgesic activity in tail immersion test in mice as compared to placebo treated mice. Additionally, anti-inflammatory function of gelsolin (8 mg/mouse) compared with anti-inflammatory drug diclofenac sodium (10 mg/kg)] was confirmed in the carrageenan injection induced paw edema where latter was measured by vernier caliper and fluorescent tomography imaging. Interestingly, results showed that plasma gelsolin was capable of reducing severity of inflammation in mice comparable to diclofenac sodium. Analysis of cytokines and histo-pathological examinations of tissue revealed administration of gelsolin and diclofenac sodium significantly reduced production of pro-inflammatory cytokines, TNF-α and IL-6. Additionally, carrageenan groups pretreated with diclofenac sodium or gelsolin showed a marked decrease in edema and infiltration of inflammatory cells in paw tissue. Our study provides evidence that administration of gelsolin can effectively reduce the pain and inflammation in mice model.

## Introduction

In today’s competitive world, people are always in race to surge ahead of each other. In doing so, we compromise with our health and suffer with pain viz. headache, back pain etc. Many of these pains are commonly due to our lifestyle and occupational hazards. In order to relieve ourselves from the pain, we rely on pain killer. Worldwide scientific research has been focused on inflammation because of its repercussion in practically all human and animal diseases. Discovery of alternative therapies for treatment of inflammation is continuing throughout the world, since many of the presently existing analgesic and anti-inflammatory drugs have numerous detrimental effects such as gastro-intestinal ulcers, bleeding, and renal disorders etc[1]. Inflammation is a defensive biological response of body to cell damage and injury to the tissue and is characterized by redness, pain, swelling, heat and loss of function at the site of injury [2,3,4,5,6,7,8,9]. Inflammation usually involves pain and edema at the site of injury due to release of many pro-inflammatory mediators [10] along with leakage of fluid from the vascular tissues due to increase in the permeability of the vessel walls, migration of inflammatory cells, tissue damage and healing.

All types of pain, whether it is acute or chronic, peripheral or central, initiate from inflammation [11]. During inflammation, many pro-inflammatory mediators such as interleukin 6 (IL-6), IL-12, interferon (INF-γ), tumor necrosis factor (TNF), cyclooxygenase-2 (COX-2) and inducible nitric oxide synthase (iNOS) are secreted [2,12].

Gelsolin (GSN), an actin-scavenging protein, is responsible for depolymerizing and capping of actin filaments, which are normally released in circulation upon cell death. For its actin activity, gelsolin is dependent on free calcium, pH and PIP2. Gelsolin can be present both as an intracellular (cytoplasmic gelsolin, cGSN) and a secreted protein (plasma gelsolin, pGSN), which are encoded by the same gene on chr 9. pGSN is 85.7 kDa protein and is identical to cGSN albeit an additional 23 amino acid sequence present in pGSN at the N- terminus, which is processed to enable its secretion [13]. Many studies have shown that pGSN levels falls by 20–50% in a wide variety of diseases such as sepsis, major trauma, acute liver injury, myocardial infarction, multiple organ dysfunction syndromes (MODS), lung injury, rheumatoid arthritis, hemodialysis, multiple sclerosis, Alzheimer’s disease, Tick-Borne Encephalitis and Lyme neuroborreliosis[14,15]. In addition, decreased levels of pGSNhave been observed in several other disease conditions that include Malaria [16], allogenic stem cell transplantation [17], hyperoxia in mice [18], oleic acid induced lung injury [19], cecal ligation/puncture model of sepsis, lipopolysacacharide (LPS, endotoxin) challenge [20,21] murine stroke [22], diabetes [23]*etc*. Currently, initial clinical trials are underway to explore the therapeutic potential of gelsolin in inflammation in humans. These are based on promising results from animal experiments, where markedly improved outcomes have been noted (for example, in sepsis, burn [24] brain inflammation [25] and diabetes in mice [23] and rats) after exogenous administration of gelsolin, thereby advocating a need for the “gelsolin replacement therapy”.

Questioning whether gelsolin repletion improves condition via analgesic mode also, we studied the analgesic and anti-inflammatory role of gelsolin in mice model of pain and carrageenan-induced paw edema. Gelsolin is generally regarded as an injury recovery protein due to its interference with fibrosis process in transgenic GSN-null mice [26]. In addition, gelsolin is reported to bind the inflammatory mediators such as platelet activating factor (PAF), lysophosphatidic acid (LPA). Thus, gelsolin acts as a buffering agent in inflammation, which by sequestering the bioactive mediators of inflammation [27,28]localizes the inflammatory and immune reactions. Although the above studies have established the role of gelsolin in inflammation; using different animal models we first time demonstrate the beneficial effects of exogenous gelsolin and propose its therapeutic role as an analgesic as well as anti-inflammatory agent.

## Materials and Methods

### Drugs, Chemicals and Reagents

Expression and purification of the recombinant human gelsolin (rhuGSN) has been described earlier [29]. For animal experiments, λ carrageenan and diclofenac sodium were purchased from Himedia, Evans Blue dye was procured from Sigma and MMPSense for Fluorescence Imaging was procured from PerkinElmer life Sciences, Waltham, MA. CBA-Flex kit for analysis of various cytokines was procured from BD Biosciences.

### Experimental Mice

Female BALB/c mice of 6–8 weeks of age were procured from the Animal Facility of the Institute of Microbial Technology (IMTECH). The protocol was approved by the Animal Ethics Committee of IMTECH, Chandigarh wide approval number IAEC/13/23. All experiments on animals were performed as per the guidelines of the Committee for the Purpose of Supervision of Experiments on Animals (CPCSEA), national regulatory body for experiments on animals, Ministry of Environment & Forests, India. In all animal experiments, mice were divided into 3 groups, each group consisting of 6 mice unless mentioned otherwise.

### Analgesic activity of gelsolin

Analgesic activity of gelsolin was evaluated using acetic acid-induced writhing test and tail immersion test in mice.

#### Acetic acid writhing test

Acetic acid-induced writhing test was performed as reported previously [30]. Briefly, mice were treated with diclofenac sodium (20 mg/kg, i.p.), a standard analgesic drug, rhuGSN and the vehicle 30 min before intra-peritoneal injection of 0.6%, 10 ml/kg body weight acetic acid. Number of abdominal constrictions i.e. writhes were counted for each group of mice starting from 5 minutes after the injection of acetic acid up to 20 minutes and expressed as percent protection. The percentage protection against acetic acid was calculated using the following formula:

$$\%\;\text{Protection}=\frac{{\text{N}}_{\text{c}}-{\text{N}}_{\text{t}}}{{\text{N}}_{\text{c}}}\times 100$$

Where N_c_ is number of writhings in control, and N_t_ is the number of writhings in test animals

#### Tail immersion test

To evaluate the analgesic activity of gelsolin, tail immersion test in mice was performed as per the method described by Aydin [31]. The lower 5 cm section of tail of mice was immersed in a beaker in which temperature of water was maintained at 55 ± 0.5°C (15). The time, in seconds, for tail withdrawal from the water was recorded as the reaction time; with a cut-off time for immersion set at 10 seconds. The reaction time was measured 30 minutes before and after intra-peritoneal administration of diclofenac sodium (20 mg/kg), the standard analgesic drug, PBS (10 ml/kg) and subcutaneous injection of rhuGSN (8 mg/mouse) and thereafter every 30 minutes up to 120 minutes.

### Anti-inflammatory activity of gelsolin in vitro and in vivo

#### Evaluation of *in vitro* anti-inflammatory activity

*In vitro anti-inflammatory activity* of gelsolin was examined by Egg Albumin Denaturation test. The reaction mixture (5 ml comprised of 0.2 ml of egg albumin, 2.8 ml of phosphate buffered saline (PBS, pH 6.4) and 2 mL of different concentrations of rhuGSN (20, 50 and 100 μg/ml). Comparable volume of double-distilled water served as control. Then the mixtures were incubated at (37°C) in a BOD incubator for 15 minutes and then heated at 70°C for 5 minutes. Following cooling, absorbance was measured at 660 nm (CECIL Aquarius CE 7500) using vehicle as blank. Diclofenac sodium at the final concentration of (20, 50 and 100 μg/ml) was used as standard drug and treated similarly for measurement of absorbance.

The percentage inhibition of protein denaturation was calculated using the following formula:

$$\%\;\text{Inhibition}=\frac{{\text{A}}_{\text{c}}-{\text{A}}_{\text{t}}}{{\text{A}}_{\text{c}}}\times 100$$

Where A_c_ and A_t_ are absorbance values in control and test samples, respectively.

#### Carrageenan-induced paw edema in mice

Carrageenan-induced paw edema in mice is a well established model of acute inflammation for screening of anti-inflammatory agents. Anti-inflammatory activity of rhuGSN was studied using carrageenan induced paw edema in mice as per previously described method [32]. Briefly, inflammation was induced in right hind paw of mice by subcutaneous injection of 0.1 ml of 1% λ carrageenan. The mice were pretreated with intra-peritoneal injection of standard drug, diclofenac sodium (10 mg/kg) and placebo (10 ml/kg) and the drug, rhuGSNsubcutaneously (8 mg/mouse) one hour before administration of carrageenan. Edema in the paw was measured at hourly interval starting from zero hour up to 5 hours using vernier caliper and compared with the controls. The inhibitory effect was determined by using following formula:

$$\%\;\text{Inhibition}=\frac{{\left({\text{C}}_{\text{t}}-{\text{C}}_{0}\right)}_{\text{control}}-{\left({\text{C}}_{\text{t}}-{\text{C}}_{0}\right)}_{\text{treated}}}{{\left({\text{C}}_{\text{t}}-{\text{C}}_{0}\right)}_{\text{control}}}\times 100$$

Where (C_t_−C_0_)_control_ is the difference in the size of paw at 5 hours in control mice, and (C_t_−C_0_)_treated_ is the difference in the size of paw at 5 hours in mice treated either with the standard drug or gelsolin.

#### Measurement of cytokines

Blood samples were collected from retro orbital plexus of mice for harvesting serum. Levels of serum cytokines such as TNF-α and IL 6 were measured by CBA-Flex kitusingBD FACSCalibur according to manufacturer’s instructions (*BD Biosciences)*.

#### Histopathology of paws

To further confirm the inflammatory changes in paws of mice after injection of carrageenan, mice were sacrificed and paws of all groups of mice were taken, fixed in 10% buffered formalin solution and stained with hematoxylin and eosin (H & E) stain to ascertain the degree of inflammation.

#### In vivo FMT tomographic imaging of carrageenan induced paws

The fluorescence imaging of inflamed paws of different groups of mice was performed with the help of the *in vivo* imager FMT 2500 Lx (Perkin Elmer Life Sciences, Waltham, MA). MMPSense 750 (cathepsin-specific activable probes) was used for visualizing inflammatory responses. The probe was injected intravenously 6 hours prior to imaging. Later on, hairs were removed by hair clipper and depilatory cream. The animals were imaged under a given laser wave length for excitation and emission fluorescence. All procedures were performed under gas anesthesia (isoflurane). The intensity of fluorescence was directly proportional to the severity of the inflammation. Image processing and analysis was performed using TrueQuant software.

#### Evans blue dye extravasations in carrageenan induced paw edema in mice

The experiments to measure increase in vascular permeability following inflammation of paw of mice were performed by Evans blue dye extravasations according to procedures described in previous studies [33,34]. Paw edema in mice was induced as described above. Mice of different groups were pre-treated with diclofenac sodium, rhuGSN and placebo as mentioned above. Evans blue dye (25 mg/kg bw) was injected into tail vein of all mice after 3.5 hr. of carrageenan injection for measuring extravasations of dye in hind paw tissues of mice as marker of inflammation. Photographs of paws of mice were taken 30 minutes after injection of Evans blue dye. After 4 hr of carrageenan injection, mice were sacrificed by cervical dislocation and paw tissues were dissected and an equal weight of paw from each mice was sliced, homogenized in 1 ml solution of acetone and 1% sodium sulfate in a ratio of 4:1 and incubated for 24 hr at 37°C to allow extraction of Evans blue from the tissues. Supernatants were collected after centrifuging the solution at 2000 rpm for 10 min and the absorbance was checked at 620 nm using a microplate spectrophotometer (PowerWave HT, Biotek). The per cent inhibitory effect of standard drug and rhuGSN in comparison to the control was calculated according to the following formula:

$$\%\;Inhibition=\frac{{\text{Abs}}_{\text{control}}-{\text{Abs}}_{\text{treated}}}{{\text{Abs}}_{\text{control}}}\times 100$$

Where Abs _control_ is the absorbance of supernatants of control mice, and Abs _treated_ is the absorbance of supernatants of paw of mice treated either with the standard drug or rhuGSN.

#### Statistical analysis

The results are expressed as Mean ± S.D. One way ANOVA followed by Dunnett and Bonferroni tests were used for analyzing the data using GraphPadInStat 3. A value of *p<0.05 was considered statistically significant (* *p*< 0.05, **p< 0.01, ***p< 0.001).

## Results

### Analgesic activity of rhuGSN

#### Effect of rhuGSN on acetic acid-induced writhing response in mice

**Fig 1:**
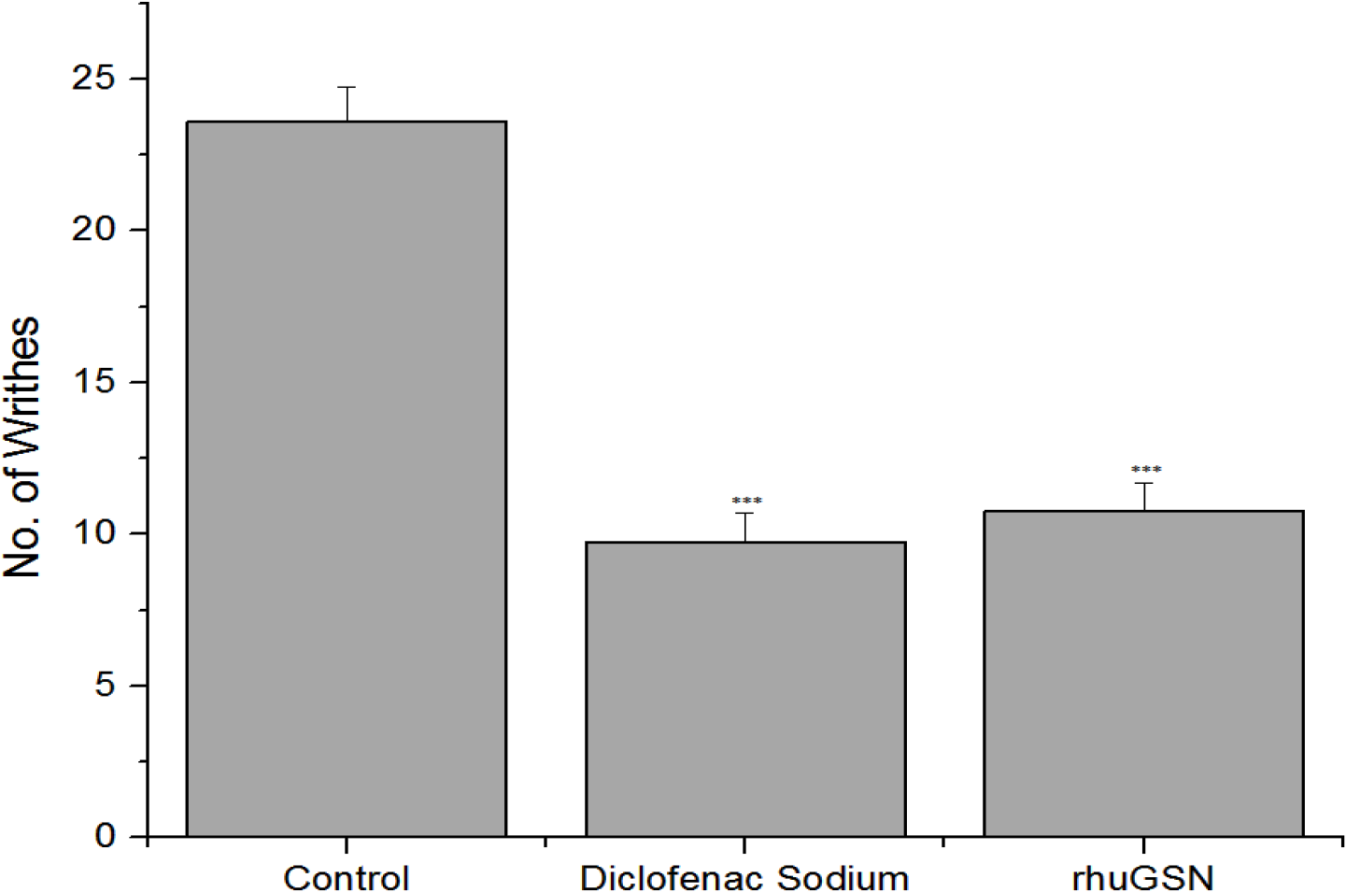
Analgesic effect of rhuGSN on acetic acid writhing in mice (N = 6).

We found that rhuGSN and diclofenac sodium significantly reduced the writhing number as compared to control mice treated with placebo only (*p* ≤ 0.01) (**Table 1**). The percentage of inhibition of writhing was 54.24 and 58.47 in rhuGSN- and diclofenac sodium-treated mice, respectively (**Fig 1**).

**Table 1:** Protective effect of rhuGSN on writhing induced by acetic acid in mice.

S. No.	Groups	Dose	No. of writhes	% Protection
**1**	Control (Saline)	0.2 ml/mouse, ip	23.67 ± 1.21	-
**2**	Standard (Diclofenac Sod.)	20 mg/kg, ip	9.83 ± 0.98***	58.47%
**3**	Test (rhuGSN)	8 mg/Mouse, sc	10.83 ± 0.98***	54.24%

#### Effect of rhuGSN on tail immersion test in mice

**Fig 2:**
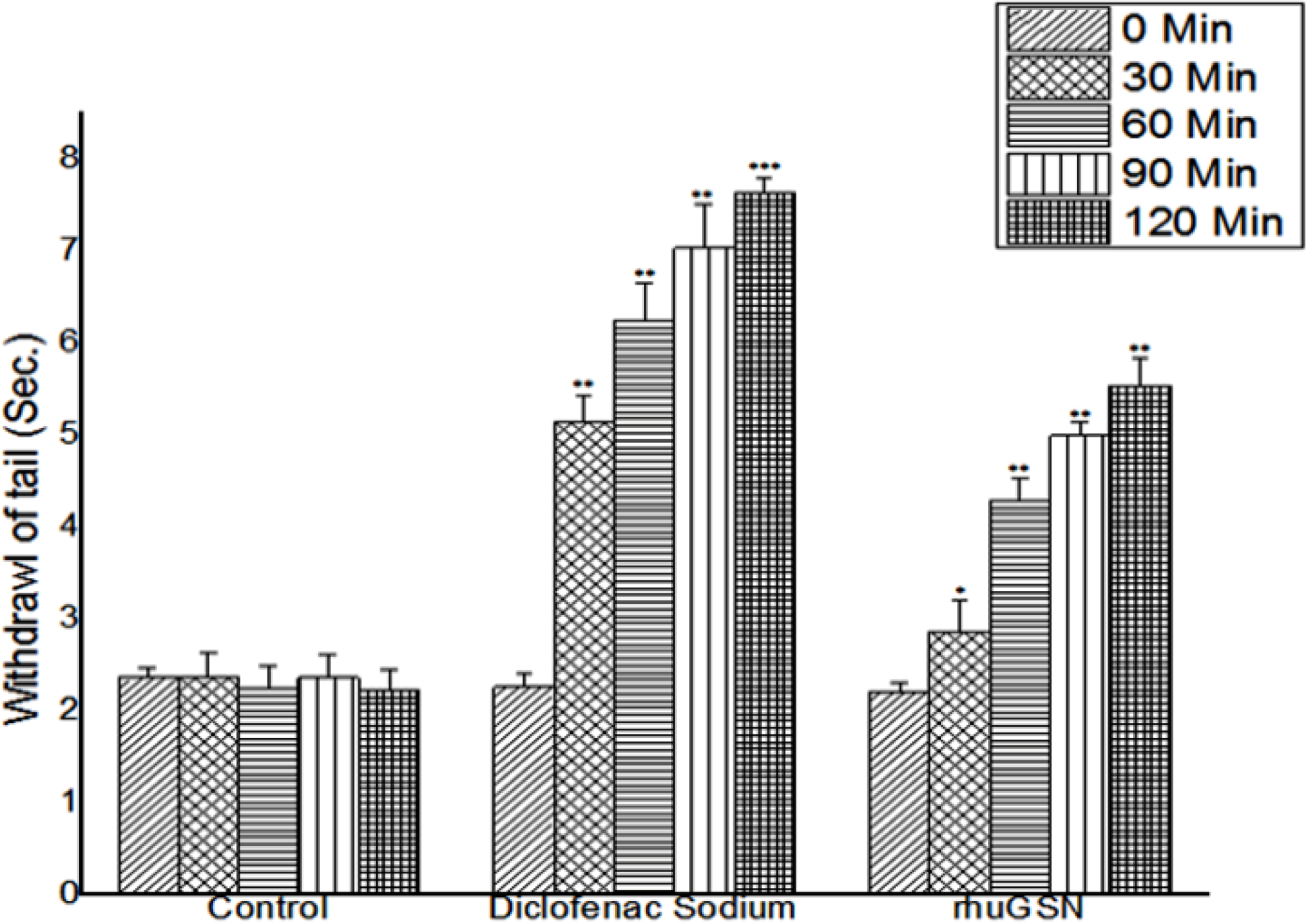
Analgesic effect of rhuGSN on tail immersion in mice (N = 6).

Tail immersion test was used to investigate the effect of central acting analgesic drugs in increasing the reaction time of mice in response to hot water. As shown in **Fig 2**, rhuGSN and diclofenac sodium produced a significant analgesic activity from 60 minutes onwards and maximum effect was observed at 120 minutes after administration of these drugs (**Table 2**). Maximum inhibition of thermal stimuli shown by rhuGSN and diclofenac sodium was 5.53 ± 0.30 and 7.64± 0.15 seconds respectively. The effect observed was statistically significant when compared with the mice of control group (p < 0.01).

**Table 2:** Analgesic effect of rhuGSN on tail withdrawal reflex induced by immersion of tail of mice in hot water.

Groups	Parameter	0 min	30 min	60 min	90 min	120 min
Control (Saline) 0.2 ml / mouse, ip	Mean Reaction Time (sec.± SD)	2.36 ± 0.11	2.36 ± 0.28	2.24 ± 0.25	2.36 ± 0.25	2.23 ± 0.22
Standard (Diclofenac Sod.) 20 mg/kg, ip	Mean Reaction Time (sec.± SD)	2.26 ± 0.14	5.14± 0.29**	6.25 ± 0.48**	7.03 ± 0.40**	7.64± 0.15***
	% inhibition	-	54.08	64.14	66.42	70.08
Test (rhuGSN) 8 mg / mouse, sc	Mean Reaction Time (sec.± SD)	2.21 ± 0.09	2.86 ± 0.35*	4.29 ± 0.15**	4.99 ± 0.23**	5.53 ± 0.30**
	% inhibition	-	17.48	44.98	52.7	59.67

### Anti-inflammatory activity of gelsolin

#### *In vitro* system

**Fig 3:**
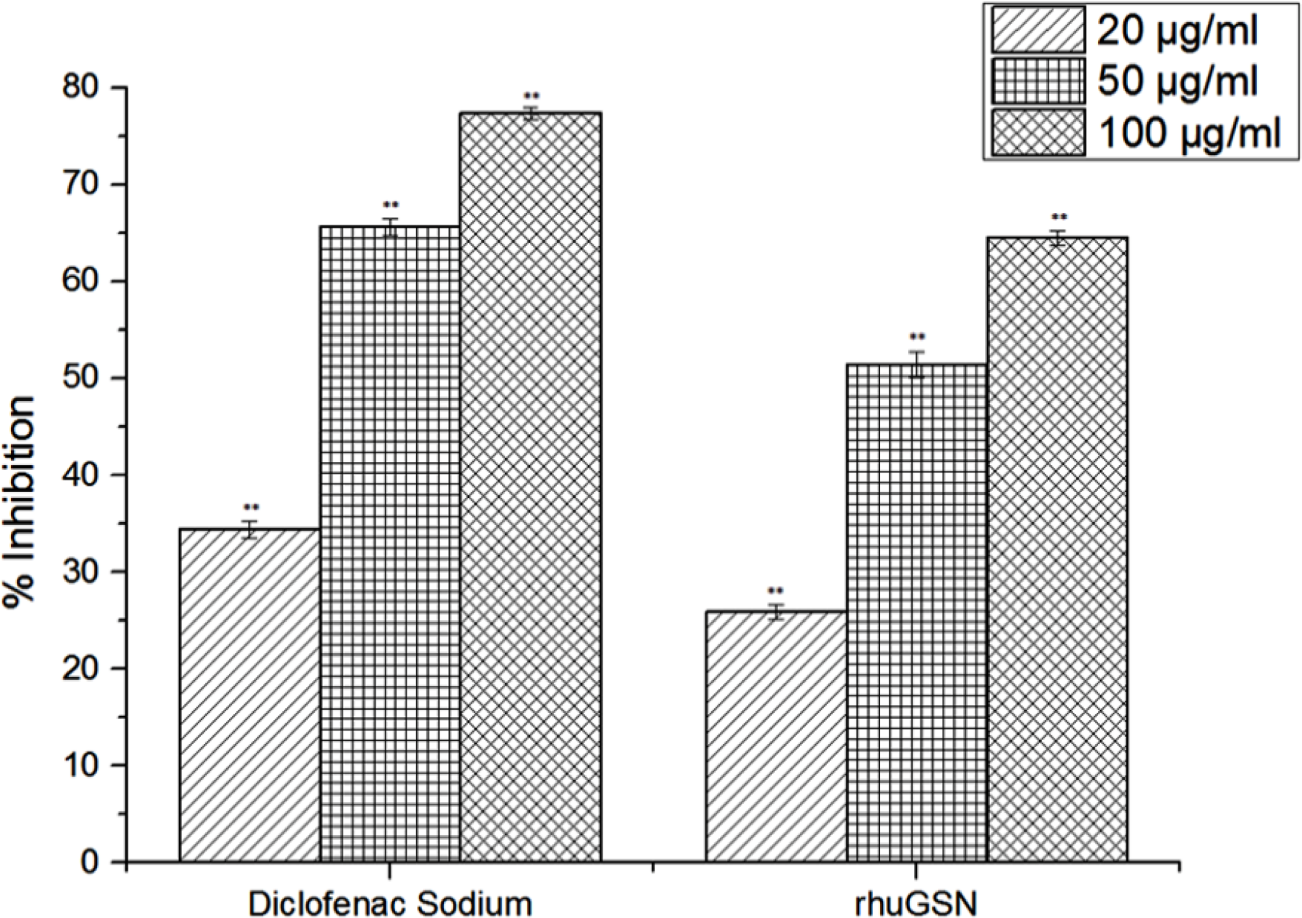
Effect of rhuGSN and diclofenac sodium on protein denaturation. Comparison curve of diclofenac sodium and rhuGSN have been plotted.

Denaturation of the tissue proteins is one of the well-documented causes of inflammation and *in vivo* denaturation of proteins has been shown in rheumatoid arthritis due to release of auto-antigens [35]. In this experiment, we explored the anti-inflammatory activity of gelsolin *in vitro* against denaturation of egg albumin induced by heat treatment. As shown in Fig 2, rhuGSN exhibited a mean inhibition of protein denaturation of 64.5, 51.4, and 25.9% for doses of 100, 50 and 20 μg/mL correspondingly, however, for diclofenac sodium these values were 77.4, 65.6 and 34.4%, respectively for similar doses (**Table 3 and Fig 3**).

**Table 3:** Effect of rhuGSN on protein denaturation *in vitro*.

S. No.	% inhibition at concentration	Control	Diclofenac sodium	rhuGSN
**1**	20 μg/ml	-	34.38 ± 0.81**	25.89 ± 0.76**
**2**	50 μg/ml	-	65.60 ± 0.83**	51.43 ± 1.29**
**3**	100 μg/ml	-	77.37 ± 0.60**	64.50 ± 0.75**

#### Carrageenan-induced paw edema in mice

**Fig 4:**
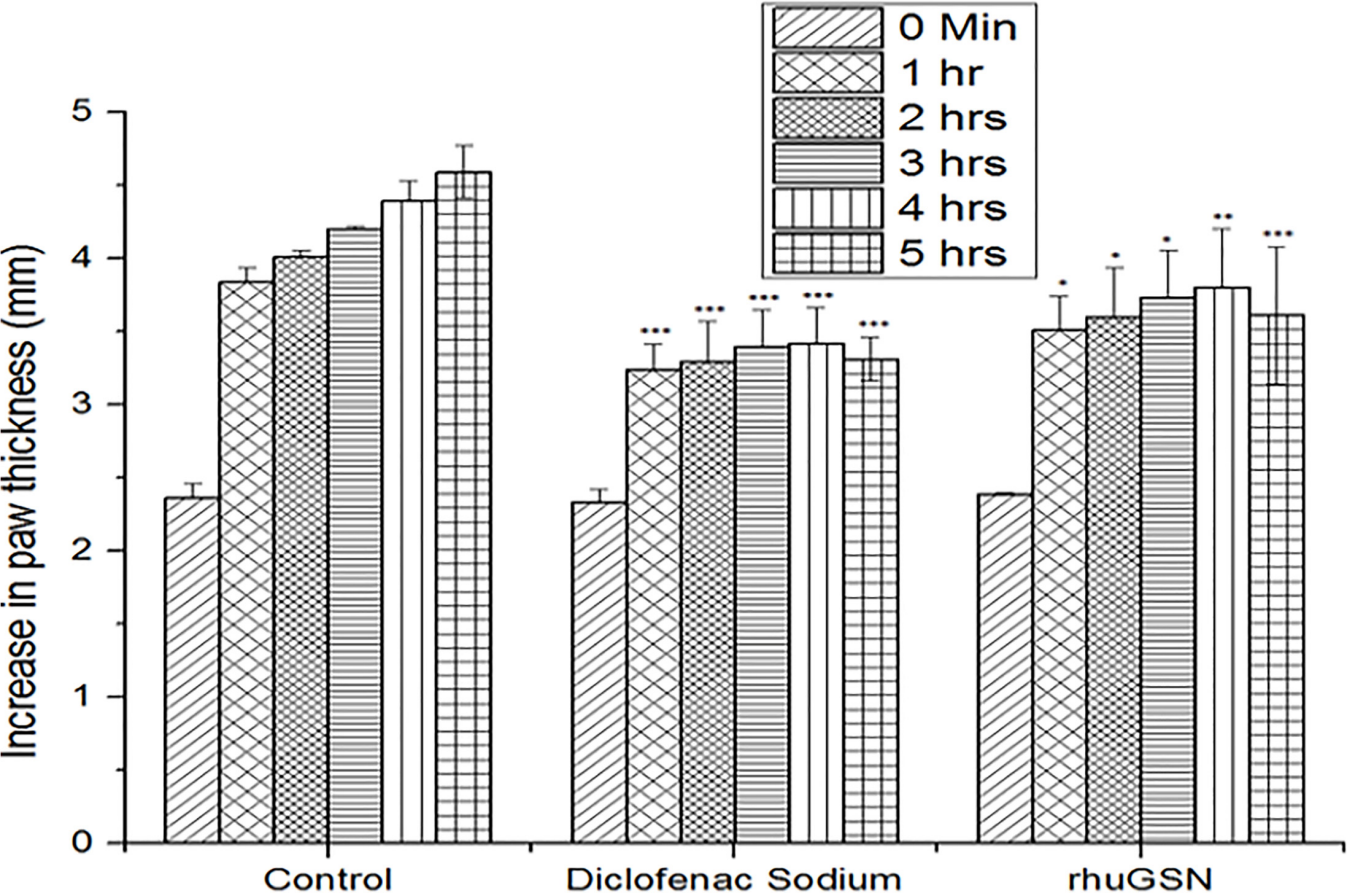
rhuGSN ameliorated the symptoms of acute inflammation induced by carrageenan. Measurement of edema of paw in mice (N = 6) of different treatment groups.

In our studies, subcutaneous injection of carrageenan caused an increase in paw size in mice due to edema, thus indicating acute inflammation of paw. **Fig 4** shows the thickness of paws in different groups of mice following treatment with rhuGSN and diclofenac sodium. Diclofenac sodium and rhuGSN exhibited 56.0% and 45.3% inhibition, respectively in comparison to the mice treated with the placebo at 5 hours (*P* ≤ 0.01) (**Table 4**).

**Table 4:** Anti-inflammatory activity of rhuGSN using carrageenan induced rat paw edema in mice.

Groups	Dose	*0 min*	*1 hr*	*2 hrs*	*3 hrs*	*4 hrs*	*5 hrs*	*% Inhibition*
Control (Saline)	0.2 ml/mouse, ip	2.36± 0.1	3.84 ± 0.10	4.01 ± 0.04	4.20 ± 0.02	4.39 ± 0.14	4.59 ± 0.18	-
Standard (Diclofenac sod.)	10 mg/kg, ip	2.33 ± 0.09	3.24 ± 0.17***	3.29 ± 0.28***	3.39 ± 0.26***	3.42 ± 0.24***	3.31 ± 0.15***	56.05
Test (rhuGSN)	8 mg/mouse, sc	2.39 ± 0.01	3.51 ± 0.23 *	3.60 ± 0.34*	3.73± 0.32*	3.80 ± 0.40**	3.61± 0.47***	45.29

**Fig 5:**
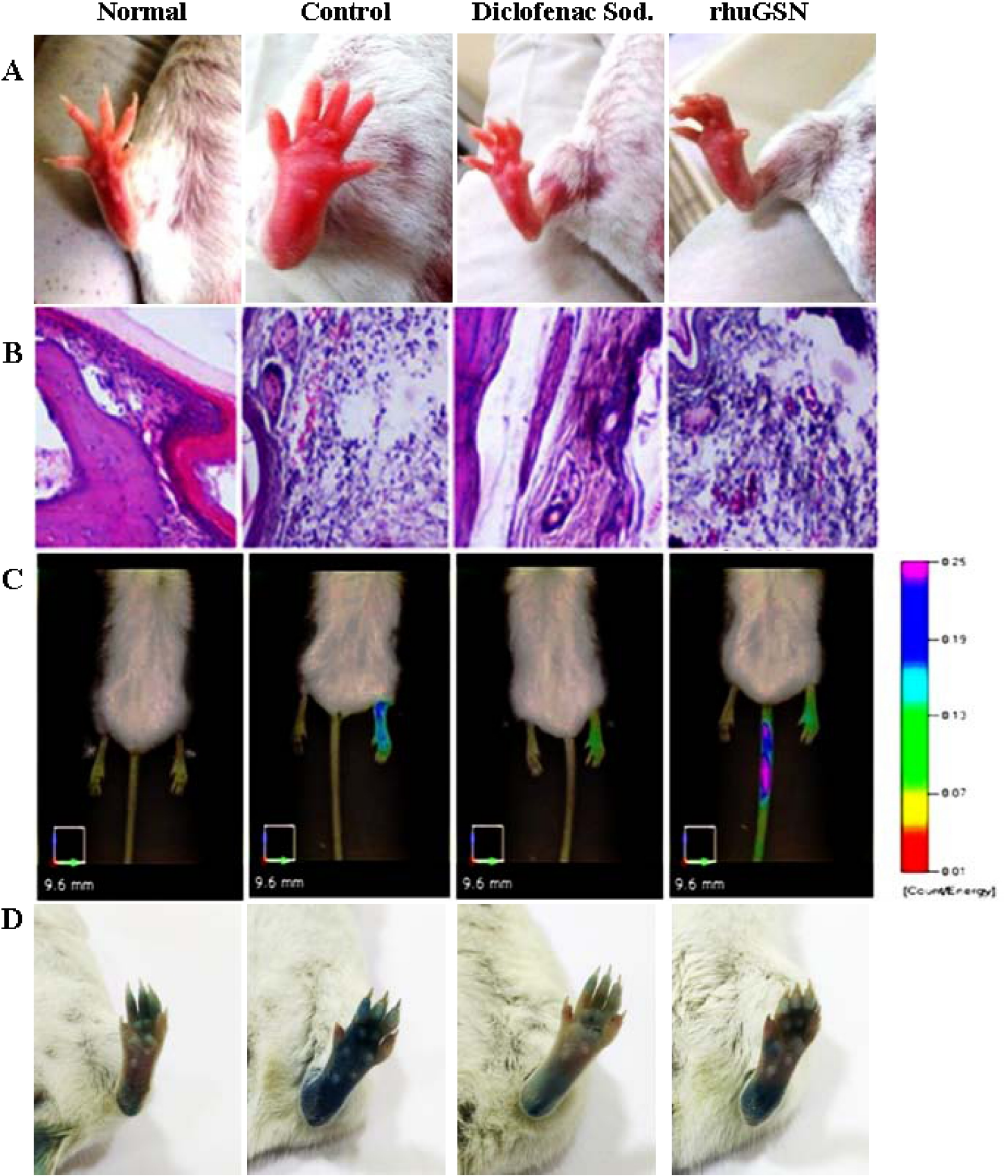
Photos of the hind paws of normal, carrageenan treated (Control), diclofenac sod. (carrageenan + diclofenac sodium) and rhuGSN (carrageenan + rhuGSN mice) (A), histopathology data representing H&E-stained sections of paws of normal, control, diclofenac sodium and rhuGSN treated mice (B), fluorescent tomographic imaging of paws (N = 3) probed with MMPSense 750, signifying a decrease in disease severity by rhuGSN treatment (C), Photos of the hind paws of normal, carrageenan treated (Control), diclofenac sod. (carrageenan + diclofenac sodium) and rhuGSN (carrageenan + rhuGSN mice) showing leakage of Evans Blue dye (D)

Photographs of the paw of various groups of mice i.e. normal control or carrageenan induced paw edema mice treated with the placebo or rhuGSN or diclofenac sodium have been shown in **Fig 5A**. These images clearly showed significant reduction in paw thickness in diclofenac sodium and rhuGSN treated groups as compared to control group.

#### Histo-pathological examination of paw

To assess the anti-inflammatory effect of rhuGSN histologically, paw tissues from all the groups of mice were examined by H&E staining. The control groups not induced with inflammation showed normal tissue. At the same time, the mice injected with carrageenan exhibited an intense edema, characterized by epithelial and conjunctive tissue blisters with substantial number of infiltrated inflammatory cells, mainly neutrophils. In contrast, carrageenan groups pretreated with diclofenac sodium (10 mg/kg) or rhuGSN (8 mg/mouse) showed a significant decrease in edema as well as decrease in the infiltration of inflammatory cells (**Fig 5B**).

#### *In vivo* FMT tomographic imaging of carrageenan induced paw

**Fig 6:**
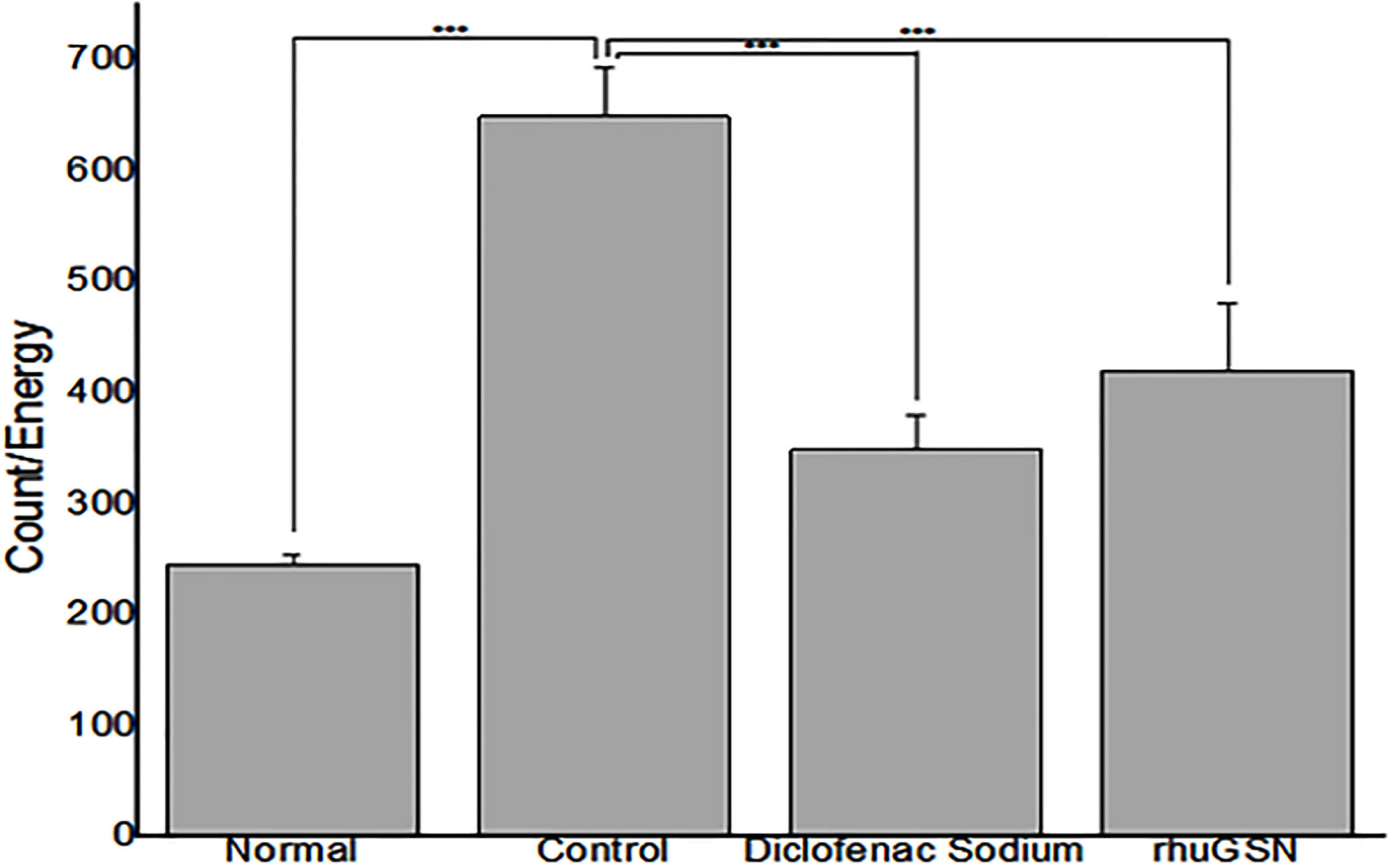
Bar diagram representing the quantification of fluorescence (inflammation) in Counts/Energy in the paws using MMPSense.

Imaging of paws of mice of carrageenan group showed maximum intensity of fluorescence, which is directly proportional to the severity of inflammation compared to control group. On the other hand, intensity of fluorescence was decreased in mice pretreated with either diclofenac sodium orrhuGSN, indicating recovery of mice from inflammation following treatment with the drugs (**Figs 5C and 6**).

#### Evans blue dye extravasation in carrageenan induced paw edema in mice

**Fig 7:**
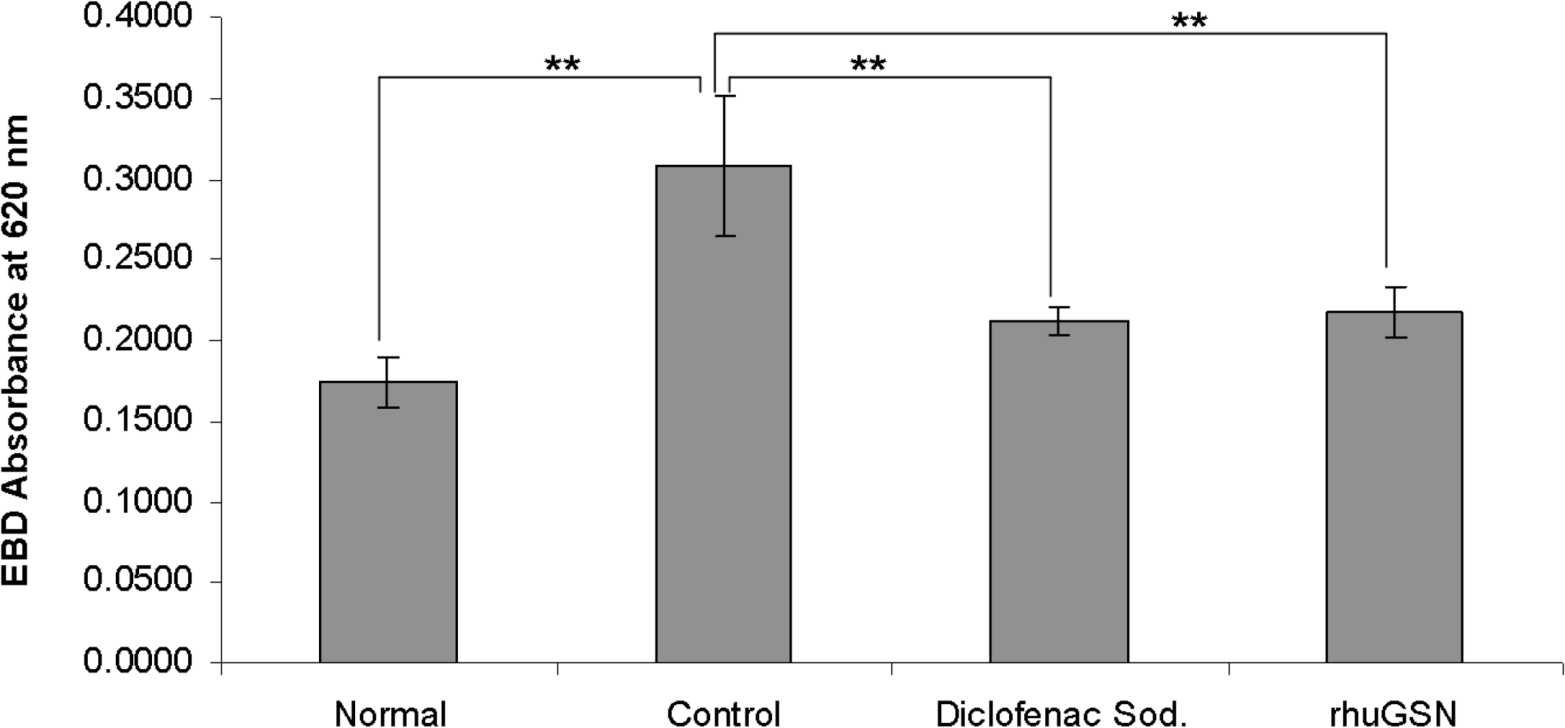
Quantification of dye extracted from carrageenan induced paw edema in mice (N = 3) following treatmentwith diclofenac sodium, rhuGSN and control.

Our results demonstrated that acute inflammation caused by carrageenan injection increases the vascular permeability, which can be adjudged by checking the evans blue extravasation in the tissues (**Fig 5D**). Absorbance data of evans blue at 620 nm clearly showed that treatment with Diclofenac sodium (10mg/kg) and rhuGSN (8mg) 1 hr prior carrageenan strikingly inhibited extravasation of Evans blue dye and exhibited 32% and 29% inhibition, respectively in comparison to the mice treated with the placebo (**Fig 7**).

#### Effect of gelsolin on expression of IL 6 and TNF-α

**Fig 8:**
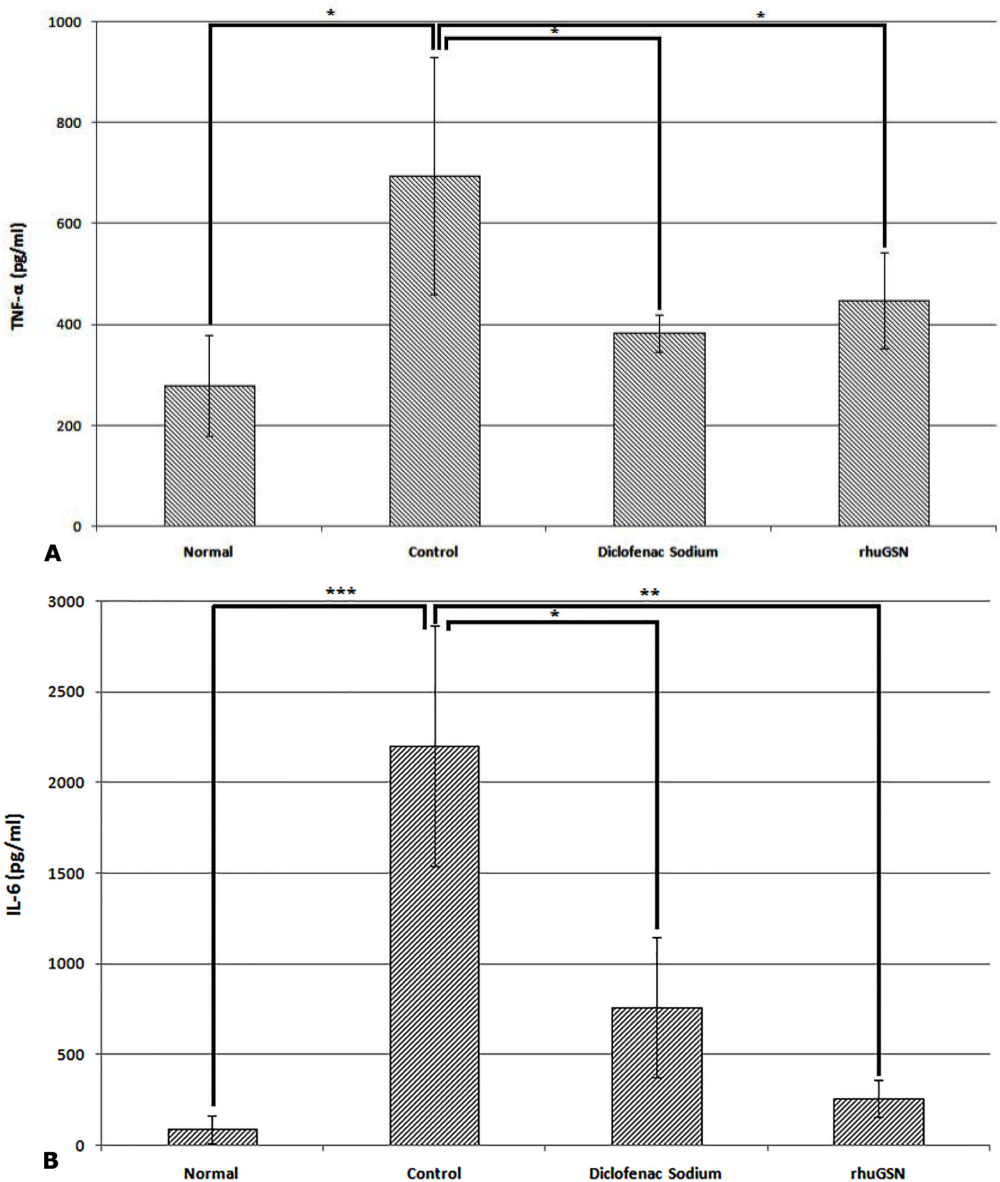
TNF-α (A) and IL-6 levels (B) in serum of mice (N = 3) are expressed as picogram/milliliter and depict the change in their levels following treatment with diclofenac sodium, rhuGSN and control.

As compared with normal mice, the levels of IL 6 and TNF-α were significantly increased (p<0.05) in the serum of mice in which acute inflammation was induced in the paw by injection of carrageenan. However, when we compared cytokine levels of control mice treated with placebo with the mice treated either with rhuGSN or diclofenac sodium, the results showed that rhuGSN and diclofenac sodium significantly (p<0.05) reduced the expression of these pro-inflammatory cytokines (**Fig 8A and 8B**).

## Discussion

Non-steroidal anti-inflammatory drugs (NSAIDs) are commonly used for treatment of pain and inflammation. However, prolonged use of these drugs leads to gastro-intestinal ulcers, bleeding, and renal disorders [1]. Thus, there is a need for discovery of new anti-inflammatory and analgesic drugs without these side effects. Gelsolin levels have been reported to decline in many diseases of humans as well as in animals [14–23] and there are several reports in the literature regarding use of gelsolin for treatment of sepsis, burn, brain inflammation, diabetes [23–25]. In this study, we have explored analgesic and anti-inflammatory activities of rhuGSN in *in-vitro* system as well as by using standard animal models. It is pertinent to mention outright that we used recombinant human gelsolin (rhuGSN) for our experiments because of the fact that mice and human GSN are 96% identical. For studying analgesic activity, we used two mice models in order to evaluate both the peripheral and central analgesic effect of rhuGSN.

Acetic acid induced writhing experiment was done to evaluate the peripheral analgesic property of test drugs [30]. It is well known that acetic acid in some way is responsible for secretion of endogenous mediators of pain thereby stimulating the neurons responsible for pain sensation, which are responsive to anti-inflammatory drugs [36]. In this study, gelsolin showed significant analgesic activity in the acetic acid-induced writhing test in mice.

In the tail immersion test, we evaluated the central analgesic property of rhuGSN. This test causes centrally mediated pain at the supra-spinal level. Increment in the response time in tail withdrawal was observed for evaluating central analgesic activity. Results of tail immersion test in our study demonstrated that administration of rhuGSN to the mice resulted in a significantly prolonged tail withdrawal reflex time in response to heat stimuli.

Taken together these two experimental results, we conclude that rhuGSN can exert both peripheral as well as central analgesic effect possibly by blocking release of endogenous inflammatory mediators like histamine, serotonin, prostaglandin *etc*. Of course, delineating the precise mechanism would be part of our future interest.

We also studied the anti-inflammatory activity of rhuGSN on protein denaturation experiments *in vitro* as well as in carrageenan-induced paw edema in mice. Denaturation of proteins has been clearly established reason of inflammation and rheumatoid arthritis and this denaturation perhaps is because of the change in electrostatic, hydrogen, hydrophobic and disulphide bonding[37]. In our experiment, rhuGSN showed significant inhibition of heat induced denaturation of protein and may possibly be the explanation of this anti-inflammatory activity.

Carrageenan-induced paw edema is generally used model for evaluating the anti-inflammatory activities of new compounds. This type of inflammation is biphasic, the initial phase is due to release of histamine, 5-hydroxytryptamine, leukotriens, kinins and cyclooxygenases in the first hour of the administration of carrageenan, and the delayed phase has been linked to production of prostaglandins, bradykinin, neutrophil infiltration etc. [38]. In present investigation, gelsolin significantly ameliorated paw edema induced by carrageenan after 5 hours. This result suggests that anti-inflammatory effect of gelsolin may be due to the inhibition of cyclooxygenase synthesis and this effect is similar to that produced by non steroidal anti-inflammatory drugs such as diclofenac sodium. However, exact mechanism how gelsolin is inhibiting cyclooxygenase synthesis would be part of our future studies.

Upon tissue injury the blood vessels in the damaged area constrict momentarily (vasoconstriction), followed by, the dilation of blood vessels (vasodilation), increasing blood flow into the area, which may last from 15 minutes to several hours. The walls of these blood vessels, which normally allow only water and salts to pass through, become permeable resulting in protein-rich fluid, called exudate, to exit into the tissues. This is followed by emigration of white blood cells into the extravascular space of the tissue. It is well established that the extravasation of fluid and proteins in interstitial spaces lead to formation of edema. Evans blue dye extravasation assay is done routinely to evaluate degree of vascular permeability following inflammation caused by injection of carrageenan [33,34,39]. In our experiment we observed that rhuGSN could significantly reduce exudation of plasma proteins in paw of mice resulting in decrease in degree of edema in comparison to the mice treated with the placebo. This experiment further confirmed the anti-inflammatory effect of gelsolin in carrageenan-induced paw edema in mice.

Several reports in the literature suggested that many cytokines for instance TNF-α, IL-1β, IL-2, IL-6, and PGE_2_ play role in during inflammation [40]. Out of these cytokines, TNF-α is most important player in inflammatory reactions, generating native protective responses by stimulating T cells and macrophages and release of kinins and leukotrienes, and further activating production of additional inflammatory cytokines [41,42]. Interleukin-6 (IL-6) is another important cytokine which is released by variety of cells at the site of injury [43]. Our data has shown that pretreatment of carrageenan mice either with rhuGSN or with diclofenac sodium reduced the TNF-α and IL-6 levels in plasma as compared to the mice treated with the placebo thus, indicating the anti-inflammatory activity of gelsolin, These results are in confirmation with earlier findings where gelsolin treatment down regulated these pro-inflammatory cytokines in sepsis, burn and inflammation of brain [24–25]. Besides these diseases, exogenous administration of gelsolin has also shown protective effect in treatment of diabetes and stroke in mice and rats [23,44].

Though these experiments evidently establishes analgesic and anti-inflammatory function of rhuGSN in these mouse models, however, the mechanism of action of pGSN of this protection remains to be explored in depth. The probable mechanism proposed in the literature involves actin depolymerization. Following any injury, actin is released from injured tissues in the circulation and gelsolin binds to this actin and after severing, remove it from the circulation and thus, limits the inflammation [20]. In addition, pGSN may also play an important role in regulating inflammation as gelsolin has been reported to act as a buffering agent in inflammation by binding to the inflammatory mediators such as platelet activating factor (PAF), lysophosphatidic acid (LPA) [27,28].

In summary, we conclude that rhuGSN can exert analgesic anti-inflammatory activity by actin scavenging from the site of injury and by down regulating the expression of pro-inflammatory cytokines such as TNF-α and IL-6.
